# Viability of human dermal fibroblasts cultured on bacterial cellulose and *Aloe vera *composites

**DOI:** 10.1186/1753-6561-8-S4-P61

**Published:** 2014-10-01

**Authors:** Lya Piaia, Camila Quinetti Paes, Luismar Marques Porto

**Affiliations:** 1 Department of Chemical Engineering and Food Engineering, Integrated Technologies Laboratory, Federal University of Santa Catarina (UFSC), Florianópolis, Santa Catarina, 88037-001, Brazil

## Background

Development of multifunctional *scaffolds *has allowed restructuration and improvement of native tissues, presenting characteristics that mimic complex tissues formation [1]. An ideal *scaffold *should provide enough transport of nutrients and also adequate mechanical support, allowing the incorporation of many cell types. Likewise, biocompatibility, mechanical properties and water retention are other important characteristics of biomaterials used for tissue regeneration [2]. In this perspective, searching for a biomaterial that promote healing and cell development, fractions of *Aloe vera *combined with bacterial cellulose (BC) are being intensively studied in our laboratory. BC hydrogels present characteristics that provide an optimal environment for cell culture [3,4]. So the combination of biological components of a natural plant and the unique properties of BC is believed to promote desirable hydrogel-cell interactions and also improvement of the healing dynamics of injured tissues [5]. The objective of this study was to evaluate the viability of human dermal cells when in contact with the novel BC-*Aloe vera *composites.

## Methods

BC-*Aloe vera *composites were developed using G. *hansenii *bacterial strain ATCC23769. The culture medium used for the production of BC membranes consisted of mannitol, yeast extract and peptone. For the composites, fractions of *Aloe vera *(gel, total gel and polysaccharide fraction 1) were added to the membranes in a concentration of 40%, at temperature of 25 ºC, under static conditions for 10 days. After that, membranes were washed with solution of NaOH (0,1 mol L^-1^), sterilized by autoclaving and stored in cool and dry place until use. In order to evaluate the cell viability, primary cultures of Human Dermal Fibroblasts adult cells (HDFa) were grown in Dulbecco's Modified Eagle Medium (DMEM) in a humidified atmosphere, at 37 ºC with 5 % of CO_2_. Groups of investigation consisted of pure BC and BC composed with fractions of *Aloe vera *extract Cell viability was determined using the colorimetric assay MTS [3-(4,5- dimethylthiazol-2-yl)-5-(3-carboxymethoxyphenyl) 2-(4-sulfophenyl)-2H-tetrazolium]. Cells were seeded in a concentration of 10^5 ^cell/membrane, grown in the porous side of the membrane and analyzed at 24, 48, 72 h and 7 days of culture. Absorbance at 490 nm was quantified in a microplate reader. In parallel to the MTS assay we also performed qualitative analysis of cell viability by Live/Dead^® ^test.

## Results and conclusions

Results indicated a good interaction between the cells and the BC composites, which was indicated by the stable cell viability observed over time. In 24 hours, we could observe an increase of cell viability in all groups when compared the control. After this time point, there was a slight decrease in cell viability, possibly caused by natural cell senescence, but remained cells were still viable. Those findings could be confirmed by the qualitative analysis (Live/Dead^®^), which showed good cell adhesion and viability over all time points. Interestingly, cell spreading was even observed at day 7. Considering these initial results, we could concluded that the formulated composites have a great potential to be used in several biomedical applications, especially for epithelial tissue repair.
